# Comparative Study of Antiproliferative Activity in Different Plant Parts of *Phaleria macrocarpa* and the Underlying Mechanism of Action

**DOI:** 10.1155/2022/3992660

**Published:** 2022-06-13

**Authors:** Yuyun Ika Christina, Muhaimin Rifa'i, Nashi Widodo, Muhammad Sasmito Djati

**Affiliations:** Department of Biology, Faculty of Mathematics and Natural Sciences, Brawijaya University, Malang 65145, East Java, Indonesia

## Abstract

*Phaleria macrocarpa* is a medicinal plant widely used in Indonesian folk medicine to treat several diseases, including cancer. However, the comparative evaluation of various plant parts of *P. macrocarpa* has not been studied for their anticancer properties on breast cancer. The study aimed to assess the antiproliferative activity of the ethanol extract of various parts of *Phaleria macrocarpa* against T47D human breast cancer cell lines. Several parts of *P. macrocarpa*, including pericarp, mesocarp, seed, and leaf, were used to determine the most potent plant part to inhibit the growth of T47D cells. The cytotoxic effects of each plant part were evaluated by WST-1 assay. The apoptotic level of T47D cells was determined by annexin V-FITC-PI and DNA fragmentation assay. Propidium iodide staining and the CFSE assay were used to examine the effect of each extract on cell cycle distribution and cell division, respectively. The relative number of caspase-3, Bax, and Bcl-2 was analyzed by flow cytometry technique. WST-1 assay revealed that *P. macrocarpa* leaves exhibited the most potent antiproliferative activity (*p* < 0.05) compared to other plant parts with selectivity only to T47D cells. *P. macrocarpa* leaves extract effectively induced apoptosis, inhibited proliferation, and arrested the cell cycle of T47D cells. The relative number of caspase-3 was significantly (*p* < 0.05) increased after being treated with *P. macrocarpa* leaf extract. *P. macrocarpa* leaf extract also leads to the dose-dependent accumulation in the Bax/Bcl-2 ratio due to upregulation of Bax and downregulation of Bcl-2. The overall results indicated that *P. macrocarpa* leaves could inhibit the proliferation of T47D cells and trigger apoptosis through caspase-3 activation and Bax/Bcl regulation. Therefore, *P. macrocarpa* leaves can be used for breast cancer therapy.

## 1. Introduction

Breast cancer is the most frequent cause of death among women worldwide, accounting for 2.1 million cases each year [1, 2]. In 2018, approximately 627,000 deaths occurred from breast cancer, accounting for at least 15% of all breast cancer incidences worldwide [3]. According to Globocan [4], approximated 58,256 breast cancer cases were diagnosed among Indonesian women in 2018, accounting for 30.5 % of cases. Breast cancer is the most challenging type of cancer that requires extensive treatment. Several common treatments for breast cancer are surgery, endocrine therapy, radiotherapy, and chemotherapy [5]. However, these treatments become increasingly ineffective due to severe side effects and drug resistance. Medicinal plant use in primary health care has increased due to their low side effect. Moreover, World Health Organization suggests using natural plant products due to their efficacy and safety [6]. Current research has focused on the novel anti-breast cancer agents, which can be effective strategies for breast cancer prevention.

*Phaleria macrocarpa* (Sheff. Boerl), widely known as Mahkota Dewa, is a native plant from Papua Island, Indonesia, that has been studied scientifically for many years. The extract of *P. macrocarpa* is reported to have valuable medicinal properties such as anti-hyperlipidemia, anti-inflammatory, anticancer, antioxidant, antibacterial, antifungal, and vasorelaxant activity [7]. Phytochemical compounds are present in different parts of this plant, such as stems, fruits, seeds, and leaves. The active compound of the different parts of this plant includes flavicordin, phalerin, gallic acid, mahkoside A, Icaricide C, dodecanoic acid, mangiferin, palmitic acid, lignans, alkaloids, and saponins [8]. The fruits of *P. macrocarpa* could reduce tumor progression by triggering apoptosis [9]. *P. macrocarpa* was shown to have an anticancer effect in breast cancer through three mechanisms, including apoptosis induction, metastasis, and proliferative inhibition [10].

This study used various parts of *P. macrocarpa,* including leaves, pericarp, mesocarp, and seeds, to determine the most potent anticancer effect among the plant part. There was limited research concerning the comparative evaluation of the anticancer properties of the various parts of this plant. Therefore, this study was focused on the in vitro anticancer investigation of the four plant parts of *P. macrocarpa* against T47D cells and the underlying mechanism of its anticancer effect.

## 2. Materials and Methods

### 2.1. Plant Materials

**Figure 1:**
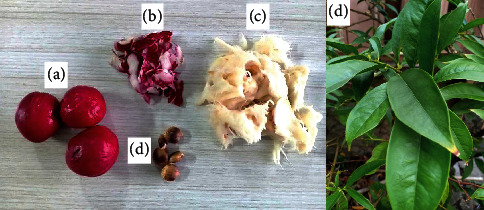
Several plant parts of *P. macrocarpa*. (a) Whole fruits; (b) pericarps; (c) mesocarps; (d) seeds; (e) leaves.

The leaves, mesocarps, seeds, and pericarps of *P. macrocarpa* were obtained in the middle of September 2019 from UPT Laboratorium Herbal Materia Medica Batu, Malang, Indonesia. *P. macrocarpa* was identified by Achmad Mabrur, SKM, M. Kes, and deposited in the UPT Laboratorium Herbal Materia Medica Batu, Malang, Indonesia, with ref no. 074/38A/102.7/2020. The morphology of different parts of *P. macrocarpa* fruit and leaves can be seen in Figure 1.

### 2.2. Preparation of Plant Extract

The extraction process is based on previous research by Djati et al. [11] with slight modifications. Each part of *P. macrocarpa* was dried for 2 days at a temperature of 50°C and then pulverized into a fine powder. A 200 g sample of each part of *P. macrocarpa* was extracted with 2 L of 95% ethanol at room temperature for 24 h in the darkroom. The samples were evaporated using a rotary evaporator (IKA® RV 10, IKA Works (Asia) Sdn Bhd, Malaysia) at 50°C. Then, each extract was kept separately in an air-tight container and stored at −20°C until being used for further analysis. Each extract was dissolved in Roswell Park Memorial Institute/RPMI-1640 (Gibco™, Thermo Fisher Scientific, USA) for T47D cells and Minimum Essential Media/MEM (Gibco™, Thermo Fisher Scientific, USA) for TIG-1 cells and then filtered with a 0.22 *µ*m syringe filter (Minisart®, Sartorius AG, Germany) before being used for treatment.

### 2.3. Cell Culture

The human breast cancer cell line T47D was obtained from the Faculty of Medicine, Brawijaya University, Indonesia. T47D cell lines were used between passage numbers 20–25. T47D cells were cultured in RPMI-1640 complete medium (RPMI-1640 supplemented with 10% fetal bovine serum/FBS (Gibco™, Thermo Fisher Scientific, USA) and 1% antibiotic (10,000 U/mL Penicillin-Streptomycin, Gibco™, Thermo Fisher Scientific, USA). Cells were incubated at 37°C in the incubator with humidified air containing 5% CO_2_. The normal human fetal lung fibroblast TIG-1 was obtained from JCRB Cell Bank (Japanese Collection of Research Bioresources Cell Bank), National Institutes of Biomedical Innovation, Health and Nutrition, Japan. TIG-1 cell lines were used between passages 10–15. TIG-1 cell lines were cultured in MEM complete medium MEM supplemented with 10% FBS and 1% antibiotic (10,000 U/mL Penicillin-Streptomycin).

### 2.4. Cytotoxicity Assay

T47D and TIG-1 cell lines (5 × 10^3^ cells/well) were seeded in 96-well plates (NEST®, NEST Scientific, USA) with RPMI and MEM complete medium, respectively, and incubated for 24 h. Cells were then treated with extracts of leaves, mesocarp, seed, and pericarp of *P. macrocarpa* with concentrations varying from 0 to 750 *μ*g/mL. After 24 h of treatment, the treatment medium was removed. Then, 10 *µ*l of WST-1 (4-[3-(4-Iodophenyl)-2-(4-nitro-phenyl)-2H-5-tetrazolio]-1,3-benzene sulfonate) reagent (Roche Diagnostics GmbH, Germany) was diluted in complete medium (1 : 10) and then added to wells and incubated for 30 min. The absorbance of the samples was measured on the BioTek ELISA Microplate Reader (BioTek Instruments, Inc., Winooski, USA) at 490 nm wavelength. The following formula determined the percentage of viability cells [12]:

(1) $$\begin{array}{c} \%\text{ cell viability}=\frac{\text{absorbance of treatment}-\text{absorbance of medium}}{\text{absorbance of control cell}-\text{absorbance of medium}}\times 100\%. \end{array}$$

IC_50_ value was calculated by a linear equation obtained from the plot between the percentages of cell viability and the log concentration of the extract. Then, the graphs were plotted in GraphPad Prism Software version 9.00 for Windows (GraphPad Software, San Diego, CA, USA).

### 2.5. Selectivity Index

The selectivity index (SI) was obtained by dividing the IC_50_ value of the *P. macrocarpa* extract into normal cells (TIG-1) by the IC_50_ value of the extract on the cancer cell line (T47D). SI value higher than three was defined as high selective extract [13].

(2) $$\begin{array}{c} \text{SI}=\frac{\text{IC}50_{\text{normal cell}}\;}{\text{IC}50_{\text{cancer cell}}\;}.\; \end{array}$$

### 2.6. Apoptosis Assay

T47D cells (3 × 10^5^ cells/well) were seeded in 24-well plates (NEST®, NEST Scientific, USA) for 24 h before treatment with IC_50_ of leaves, mesocarp, seed, and pericarp of *P*. *macrocarpa*. Cisplatin (PT. Ferron Par Pharmaceuticals, Indonesia) was used as a positive control. After 24 and 48 h of treatment, cells were washed with phosphate-buffered saline/PBS (Biowest, USA) twice and harvested by adding 75 *μ*l trypsin EDTA. Cells were stained with FITC Annexin V/propidium iodide (PI) (BioLegend™, San Diego, USA). 5 *μ*L of annexin V-FITC-PI was added to the cell. The suspensions were mixed and incubated for 20 min in darkness at room temperature. The level of early apoptotic cells (annexin V^+^/PI^−^), late apoptotic cells (annexin V^+^/PI^+^), necrotic cells (annexin V^−^/PI^+^), and live cells (annexin V^−^/PI^−^) was analyzed using a flow cytometer (BD FACSCalibur™, San Jose, CA) with FlowJo™ v.10 Software (Vancouver, BC). Each experiment was conducted in triplicate.

### 2.7. Morphological Assessment

Briefly, T47D and TIG cells were seeded in 24-well plates and incubated with IC_50_, 2x IC_50_ (2-fold of IC_50_), and 4x IC_50_ (4-fold of IC_50_) of each plant part of *P. macrocarpa* for 48 and 72 h. Cells were visualized with an inverted microscope (Olympus, Center Valley, PA, USA) with 200x magnification.

### 2.8. DNA Ladder Assay

T47D cells were (3 × 10^5^ cells/well) seeded in 24-well plates for 24 h prior and then treated with IC_50_, 2x IC_50_ (2-fold of IC_50_) and 4x IC_50_ (4-fold of IC_50_) of each extract. After 24 and 48 h of treatment, cells were harvested and transferred to a 1.5 mL tube. Then, the suspension of the cells centrifuges at 13,000 rpm. The obtained pellet was resuspended with 200 *µ*l PBS. The NEXprep™ Cell/Tissue DNA Mini Kit (NEX Diagnostics, Genes Laboratories, Korea) protocol was used to isolate the genomic DNA of T47D cells. The obtained DNA of T47D cells from each treatment was subjected to electrophoresis in TBE buffer on 1.5% agarose, 100 volts, 60 min. 1 kb DNA ladder (Promega Corporation, USA) was used as a marker. The fragmented DNA bands were imaged in a cooled CCD imager (ImageQuant™ LAS 500, USA).

### 2.9. Cell Division Assay

The effect of the extracts on cell division was determined by CFSE (carboxyfluorescein succinimidyl ester) assay (CellTrace™, Thermo Fisher Scientific, USA). CFSE was usually used to detect cell proliferation by measuring CFSE quantities in the cells. Briefly, T47D cell lines were added with 5 *μ*M of CFSE at 37°C for 20 min, followed by 500 *μ*l PBS. Cells were then washed with 500 *μ*l of FBS. The suspension cells were washed with PBS twice. Then, CFSE-stained cells (3 × 10^5^ cells) were seeded in 24-well plates. After 24 and 48 h, cells were treated with IC_50_ of each extract of *P. macrocarpa* and IC_50_, 2x IC_50,_ and 4x IC_50_ of *P. macrocarpa* leaves. The cell division was determined using flow cytometer (BD FACSCalibur™, San Jose, CA) with FlowJo™ v. 10 Software (Vancouver, BC). Each experiment was performed in triplicate.

### 2.10. Cell Cycle Analysis

T47D cells (3 × 10^5^ cells/well) were seeded in 24-well plate for 24 h and then treated with IC_50_ of each extract of *P. macrocarpa* and IC_50_, 2x IC_50_, and 4x IC_50_ of *P. macrocarpa* leaves in a humidified air of 5% CO_2_ at 37°C. After, 24 and 48 h of treatment, cells were trypsinized, added with 70% ethanol, and incubated for 30 min. PBS was used to wash the cells twice. Propidium iodide (10 *μ*g/mL) was added and incubated for 20 min. A total of 10.000 events per sample was measured using a flow cytometer (BD FACSCalibur™, San Jose, CA) with BD Cell Quest Pro software. Each experiment was performed in triplicate.

### 2.11. Analysis of Caspase-3, Bax, and Bcl-2 Levels

Cells were harvested after 24 h treatment with *P. macrocarpa* leaves (IC_50_, 2x IC_50_, and 4x IC_50_) using trypsinization. After centrifugation for 5 min at 2500 rpm, 100 *μ*l of cell suspension was placed in 1.5 mL microtube. Fifty *μ*l of intracellular fixation buffer (eBioscience™, Thermo Fisher Scientific, USA) was added into each tube and incubated for 20 min in a cool box. After incubation, each tube was added with 500 *μ*l of diluted (10-fold in distilled water) Permeabilization Buffer (eBioscience™, Thermo Fisher Scientific, USA) and then centrifuged at 2500 rpm for 5 min. Pellets were then stained with the following combination antibody: anti-caspase-3 p11 (C6) (HRM)-Alexa Fluor 647, Bax (2D2): sc-20067 (HRM)-PerCp-Cy5.5, and Bcl-2 (C-2): sc-7382 (HRM)-PerCp-Cy5.5 (Santa Cruz Biotechnology, Inc., Texas, USA). 15.000 events per sample were measured using a flow cytometer (BD FACSCalibur™, San Jose, CA) with FlowJo™ v. 10 Software (Vancouver, BC).

### 2.12. Statistical Analysis

The percentage of apoptosis, cell division, and cell cycle phase was further analyzed using two-way ANOVA followed by Tukey's test. The relative number of Bcl2, Bax, and caspase-3 was statistically analyzed using one-way ANOVA followed by Tukey's test. All statistical analysis was performed using IBM SPSS Statistics for Windows ver. 22.0 (IBM Corp., Armonk, NY). Data were expressed as mean ± standard deviation (SD). *p* < 0.05 was indicated to be statistically significant.

## 3. Results

### 3.1. Cell Viability of T47D and TIG-1 Cells

**Figure 2:**
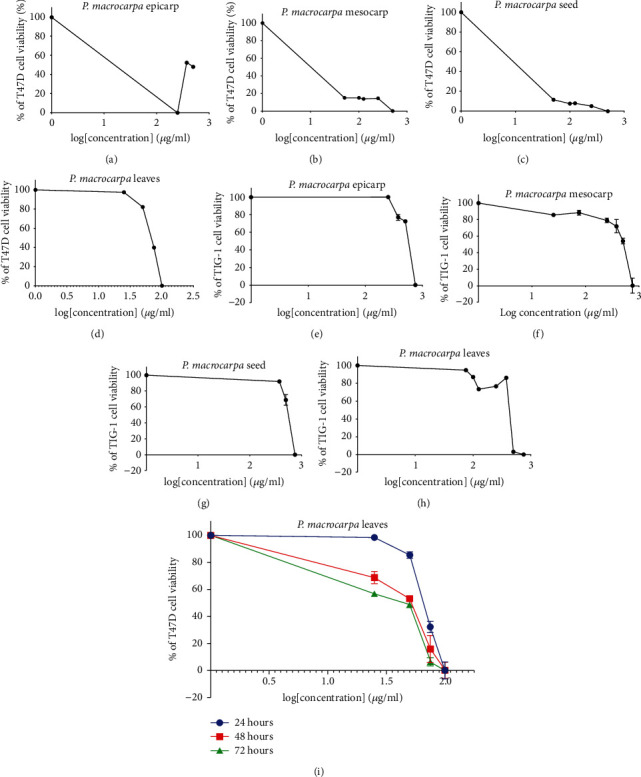
Effect of ethanol extract of various parts of *P. macrocarpa* on T47D and TIG-1 cell viability. T47D cells were treated with ethanol extract of (a) pericarp; (b) mesocarp; (c) seed; (d) leaves for 24 h TIG-1 cells treated with ethanol extract of (e) pericarp; (f) mesocarp; (g) seed; (h) leaves for 24 h; (i) T47D cells treated with ethanol extract of leaves at the different durations of treatment (24, 48, and 72 h as measured by WST-1 assays. Data are shown as mean ± SD of three independent experiments.

The results demonstrated that the ethanol extract of all parts of *P. macrocarpa* exerted a cytotoxic effect on T47D cells. The ethanol extract of pericarp, mesocarp, seed, and leaves could decrease the viability of T47D cells with IC_50_ values at 24 h calculated as 125, 144, 139, and 97 *μ*g/mL, respectively (Figure 2(a)–2(d)). The ethanol extract of pericarp, mesocarp, and seed showed the lowest cytotoxicity activity due to an IC_50_ value greater than 100 *μ*g/mL. Interestingly, only leaf extract showed promising in vitro cytotoxic activity against T47D cells due to an IC_50_ value of fewer than 100 *μ*g/mL.

Further investigation was carried out to determine the cytotoxicity activity of *P. macrocarpa* leaf extracts at different incubation times. *P. macrocarpa* leaf extract decreased the viability of T47D cells in a dose- and time-dependent manner.  Figure 2(i) shows the dose-dependent growth inhibitory effects of *P. macrocarpa* leaf extract on the cell viability of T47D cells at 24, 48, and 72 h with IC_50_ values of 97, 42, and 33, respectively. Interestingly, a robust antiproliferative effect of *P. macrocarpa* leaf extract was only observed on the breast cancer cell lines but was less toxic to normal fibroblast cells (TIG-1), indicated by an SI value greater than 3 (Table 1). All extracts did not exert a cytotoxic effect on TIG-1 cells (Figures 2(e)–2(h)).

### 3.2. Apoptosis Level in T47D Cells

**Figure 3:**
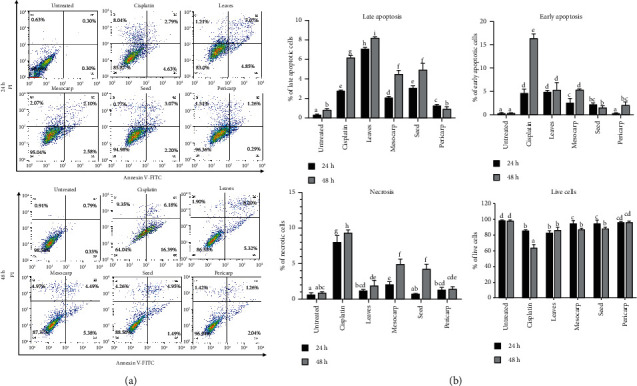
Ethanol extract of *P. macrocarpa* leaves triggered more apoptosis of T47D cells. (a) Dot plot diagrams showed the percentage of T47D cell populations in different stages (necrosis, early apoptosis, late apoptosis, and live cells) after being treated with IC_50_ value of each part of *P. macrocarpa* extract for 24 and 48 h and evaluated by annexin V-FITC/PI and flow cytometry analysis. (b) The representative bar showed the percentage of cells undergoing apoptosis. The value was expressed as mean ± SD (*n* = 3). The different subsets indicated statistical differences (*p* < 0.05). Necrotic cells (upper left quadrant: annexin V^−^PI^+^); late apoptotic (upper right quadrant: annexin V^+^PI^+^); early apoptotic (lower right quadrant: annexin V^+^PI^−^); and live cells (lower left quadrant: annexin V^−^PI^−^).

The results showed that leaves, mesocarp, seed, and pericarp of *P. macrocarpa* significantly (*p* < 0.05) induced late apoptotic cells at 24 h compared to the untreated control group (7.07, 2.10, 3.07, and 1.26 % versus 0.30%) (Figure 3(a)). In contrast to the other treatment groups, treatment with IC_50_ of *P. macrocarpa* leaf extracts significantly increased (*p* < 0.05) the percentage of late and early apoptotic T47D cells in a time-dependent manner (Figure 3(b)). Furthermore, IC_50_ of *P. macrocarpa* leaves exhibited lower necrosis cell levels than the cisplatin group (*p* < 0.05). The percentage of T47D live cells was also markedly decreased in IC_50_ of *P. macrocarpa* leaf group compared to the untreated group.

### 3.3. Morphological Changes of T47D Cells

**Figure 4:**
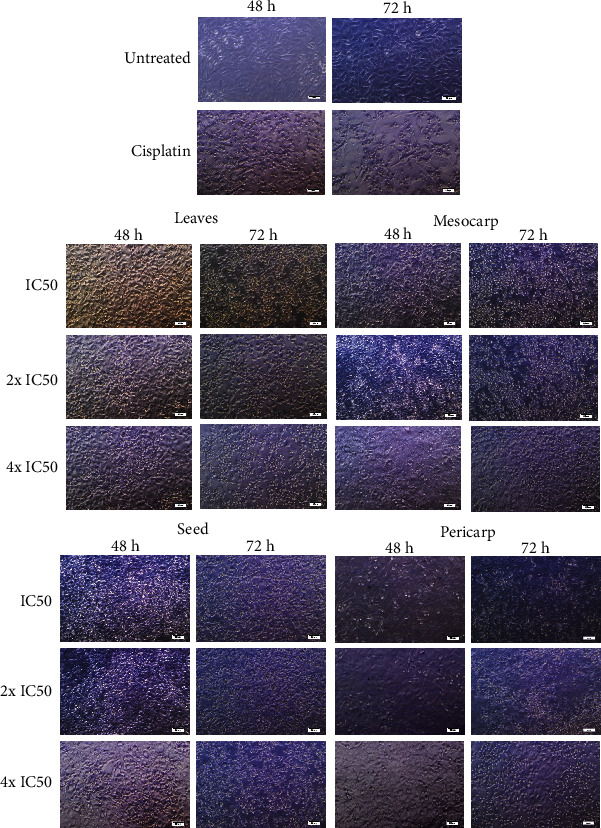
Representative photomicrograph showed morphological changes in T47D cells after being treated with ethanol extract of each part of *P. macrocarpa* at IC_50_, 2x IC_50_ (2-fold of IC_50_), and 4x IC_50_ (4-fold of IC_50_).

The observation showed that the untreated control group maintained their normal morphology (Figure 4). Treatment with various parts of *P. macrocarpa* exhibited morphological changes and apoptotic features of T47D cells, including rounding, shrinkage, and membrane blebbing. When the concentration was increased, T47D cells undergoing apoptosis indicated by several apoptotic features such as apoptotic bodies and decreased cell number. *P. macrocarpa* leaf extract exhibited greater morphological changes and decreased live cells than another group based on photomicrograph visualization.

### 3.4. DNA Laddering Results

**Figure 5:**
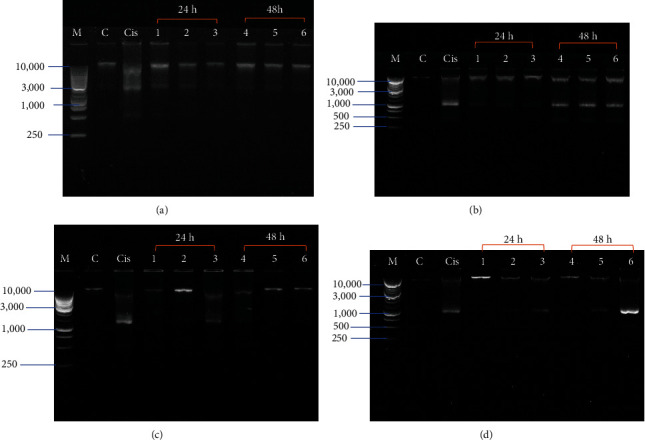
DNA fragmentation of T47D cells treated different parts of *P. macrocarpa* for 24 and 48 h. (a). Pericarp; (b) seed; (c) mesocarp; (d) leaves. From left lane: M (DNA ladder 1 kb), C (untreated control cells), Cis (treated cells with 1 *µ*g/mL of cisplatin), Lane 1–3 (treated cells with different concentrations (IC_50_, 2x IC_50_/2-fold of IC_50_, and 4x IC_50_/4-fold of IC_50_) for 24 h, and Lane 4–6 (treated cells with different concentrations (IC_50_, 2x IC_50_/2-fold of IC_50_, and 4x IC_50_/4-fold of IC_50_) for 48 h.

As the four extracts of the different parts of *P. macrocarpa* showed cytotoxicity effect on T47D cells, we further investigated DNA fragmentation as a marker of late apoptosis using DNA ladder assay. Based on Figure 5, treating T47D cells with all part of *P. macrocarpa* produces DNA fragmentation. DNA fragmentation was determined by the formation of the typical laddering pattern. The band of fragmented DNA was found in all treatment group at a range of 1,000–500 bp, as demonstrated in Figure 5. Nonfragmented DNA was found in the untreated control group, which was indicated by a single band in the agarose gel at 10.000 bp.

### 3.5. *P. macrocarpa* Leaves Reduce More Cell Division

**Figure 6:**
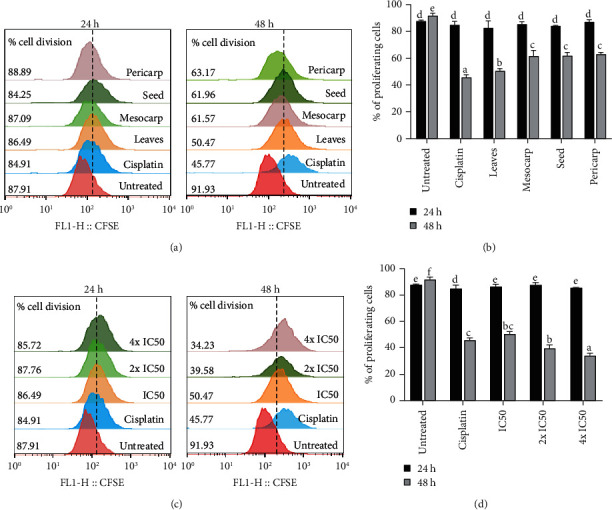
Effect of ethanol extract of various parts of *P. macrocarpa* on T47D cell division. The fluorescence intensity of carboxyfluorescein succinimidyl ester (CFSE) was recorded by flow cytometry. (a, b) Overlay histogram and the representative graph showed the percentage of cell division after treatment with all extracts of *P. macrocarpa* for 24 and 48 h. (c) Further investigation in cell division after 24 and 48 h treatment with *P. macrocarpa* leaves with three different concentrations (IC_50_, 2x IC_50_/2-fold of IC_50_, and 4x IC_50_/4-fold of IC_50_). (d) The representative graph demonstrated the percentage of cell division in all treatment group. Data are expressed as mean ± SD of three independent experiments (*p* < 0.05). The significant result (*p* < 0.05) was indicated by a different subset.

As assessed by CFSE assay, T47D cell lines were strongly affected by treatment with ethanol extract of leaves, mesocarp, seed, and pericarp of *P. macrocarpa* after 48 h (Figure 6(a)). The percentage of cell division was still high at 24 h in all treatment groups. At 48 h of treatment, all extracts exhibited a significant reduction (*p* < 0.05) in the cell division compared to the untreated group (50.47, 61.57, 61.96, 63.17 %, respectively, versus 91.93 %). Interestingly, the highest reduction in cell division was found in *P. macrocarpa* leaf extract and cisplatin group after 48 h treatment. We further demonstrated that the highest concentration of *P. macrocarpa* leaves (4x IC_50_ or 388 *µ*g/mL) could reduce cell division more elevated than the positive control, cisplatin (Figure 6(b)), at 48 h of treatment. The alteration of cell division was clearly observed by an overlay histogram (Figure 6(c)).

### 3.6. *P. macrocarpa* Leaves Induce Cell Cycle Arrest in T47D Cells

**Figure 7:**
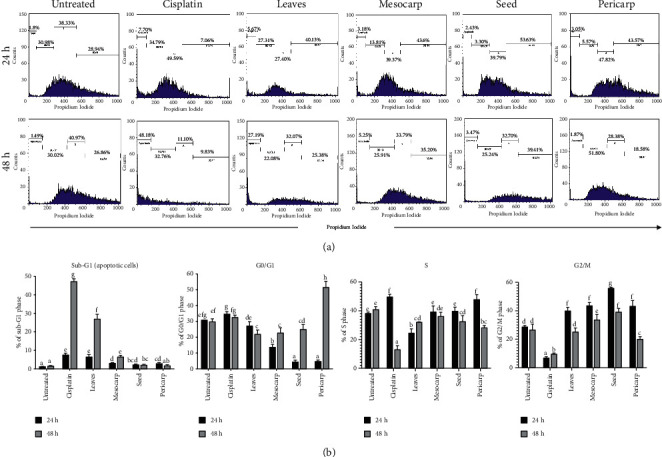
Evaluation of cell cycle of T47D cells treated with various parts of *P. macrocarpa* (IC_50_ concentration) for 24 and 48 h. (a) The histogram showed the distribution of the cell cycle phase. (b) The representative bar showed the percentage of each cell cycle phase from each sample. The values are presented as mean ± SD of three independent plates. A different subset indicated a significant result (*p* < 0.05).

To investigate how the cell cycle distribution of T47D cells is affected by the various parts of *P. macrocarpa*, the DNA content analysis was carried out using PI staining. Histogram of the cell cycle phase illustrated that the T47D cells were categorized into sub-G0 or apoptosis, G0/G1, S, and G2/M phases (Figure 7(a)). At 24 and 48 h, a notable accumulation of cells in G0/G1 (apoptotic cells), S phases, and the number of proliferating cells (G2/M) were observed in the untreated control group (Figure 7(b)). The leaves, mesocarp, and seed extract of *P. macrocarpa* have effectively reduced the proportion of G0/G1 at 24 h and 48 compared to the untreated group. Treatment with pericarp extracts significantly accumulated S phase at 24 h (47.82) and G0/G1 at 48 h (51.80%). These data are related to a low apoptotic cell observed in *P. macrocarpa* pericarp extract. Besides, IC_50_ of *P. macrocarpa* leaf extract significantly arrested the proliferating cell, which indicated a high number of sub-G0/G1 at 48 h compared to mesocarp, seed, and pericarp group (27.19 % versus 5.25%, 3.47%, 1.87%, respectively). Furthermore, the cisplatin group exhibited a high sub-G0/G1 phase at 48 h, indicating that cisplatin induces apoptotic cells more than leaf extract (48.18 % versus 27.19).

**Figure 8:**
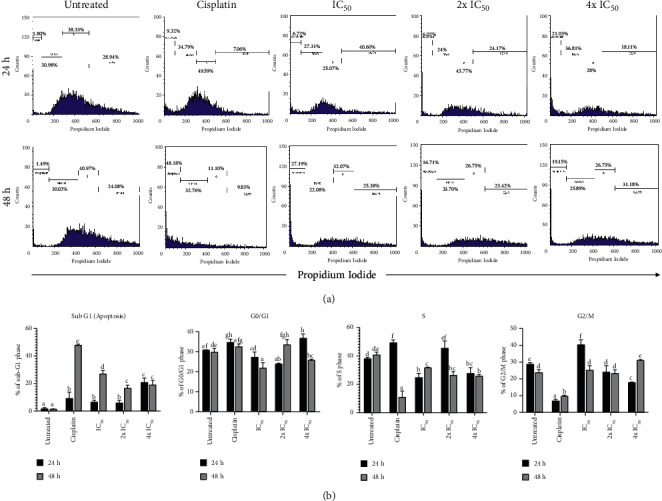
*P. macrocarpa* leaves induce cell cycle arrest of T47D cells. (a) T47D cells were treated with three different concentrations (IC_50_, 2x IC_50_/2-fold of IC_50_, and 4x IC_50_/4-fold of IC_50_) of *P. macrocarpa* leaf extract, followed by PI staining and analyzed using flow cytometer. The cell cycle distribution pattern was represented by a histogram. (b) The representative bar showed the percentage of each cell cycle phase from each sample. Data are presented as mean ± SD (*n* = 3). The significant result (*p* < 0.05) was indicated by a different subset.

Next, we investigate whether different concentrations and durations of *P. macrocarpa* leaf treatment could induce a high cell cycle arrest of T47D cells. We performed cell cycle analysis in T47D cells treated with IC_50_, 2x IC_50_, and 4x IC_50_, and a comparative analysis of the percentage cells of each phase was observed at 24 and 48 h (Figure 8). When the cells were treated with all concentrations of the *P. macrocarpa* leaf extract, a significant elevation in the sub-G1 phase was generally demonstrated in a time- and dose-dependent manner, followed by S phase reduction. The study found that IC_50_ of *P. macrocarpa* leaves causes a significant decline in the number of cells in the G0/G1 phase (*p* < 0.05) and a reduction in the S phase at both time points observed. A G0/G1 phase accumulation was found in 2x and 4x IC_50_ of *P. macrocarpa* leaf extract, which was more significant in high concentration (4x IC_50_) at 24 h. The increased accumulation of sub-G0 phase was also found in the IC_50_ group than two other concentrations at 48 h (27.19 % versus 16.71 and 19.15 %, respectively). IC_50_ of *P. macrocarpa* was found to arrest the cell cycle of T47D cells in the S phase, followed by an accumulation of cells undergoing apoptotic cell death or in the sub-G1 phase.

### 3.7. *P. macrocarpa* Leaf Extract Mediated Caspase-3 Activation and Bax/Bcl-2 Ratio

**Figure 9:**
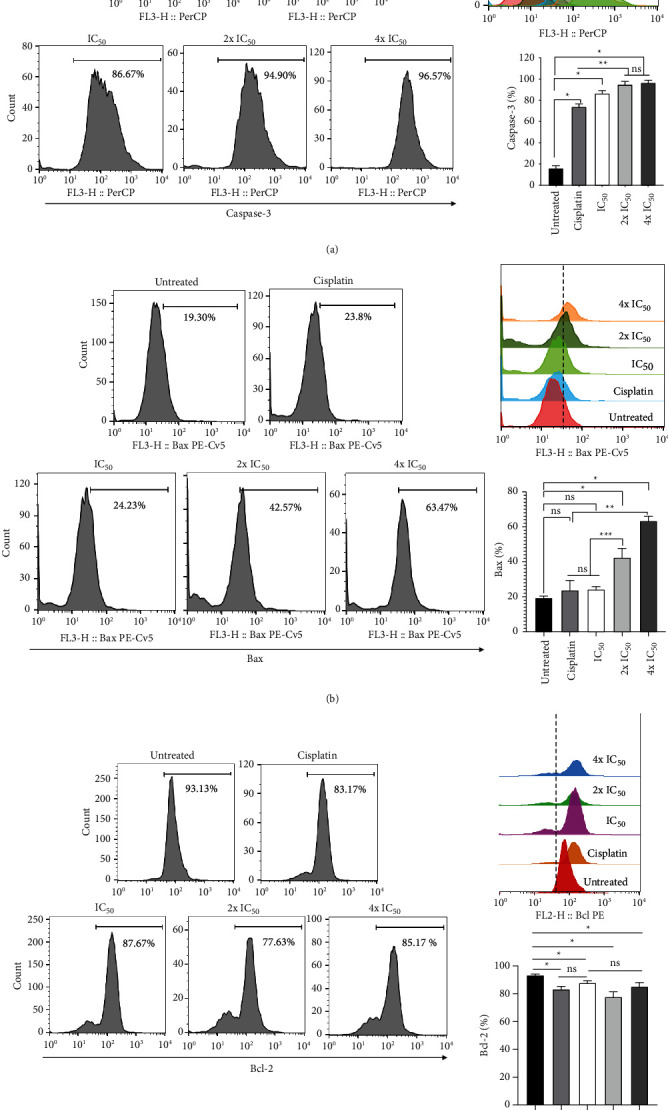
Effect of *P. macrocarpa* leaf extract on the relative number of caspase-3, Bax, and Bcl-2 in T47D cells as assessed by flow cytometry analysis. T47D cells were treated with *P. macrocarpa* leaf extract at IC_50_, 2x IC_50_/2-fold of IC_50_, and 4x IC_50_/4-fold of IC_50_ for 24 h. The representative histogram and graph showed the percentage of T47D cells which expressed (a) caspase-3, (b) Bax, and (c) Bcl-2. Data are presented as mean ± SD (*n* = 3). *p* < 0.05. ^*∗*^: significant compared with the untreated control group; ^*∗∗*^: significant compared with cisplatin group; ^*∗∗∗*^: significant compared with cisplatin and IC_50_ group; ns: no significant difference.

To investigate the mechanism of *P. macrocarpa* leaf extract to trigger apoptosis, the expression of the pro-apoptotic and anti-apoptotic protein was analyzed by flow cytometry. Caspase-3 is a protein responsible for triggering apoptosis. When compared to the untreated control group, all group treatments of *P. macrocarpa* leaves significantly (*p* < 0.05) activated caspase-3 in a dose-dependent manner (15.9 % versus 86.67 %, 94.90 % and 96.57 %, respectively) (Figure 9(a)). Cisplatin group also could increase the level of caspase-3 (74.07 %). However, the percentage is lower than *P. macrocarpa* leaf extract treatment.

The level of Bax also significantly increased, while the level of Bcl-2 declined after treatment with *P. macrocarpa* leaf extract (Figure 9(b)). The level of Bax expression was raised in a dose-dependent manner (24.23 %, 42.57%, and 63.47 %, respectively) compared to untreated control (19.30 %) (Figure 9(c)). *P. macrocarpa leaf* treatment also leads to the dose-dependent increase in the Bax/Bcl-2 ratio due to upregulation of Bax and downregulation of Bcl-2, which contributes to *P. macrocarpa* leaves-induced apoptosis (Figure 9(d)).

## 4. Discussion

Traditional herbal medicine has been utilized for treating and curing several diseases. Nowadays, herbal remedies are interested in discovering novel drugs for cancer cases. The herbal remedies commonly use a natural compound of the plant, which produces biological activity to treat disease [14]. This study evaluated the anticancer activity of four parts of *P. macrocarpa,* including leaves, pericarp, mesocarp, and seed against T47D cells. The use of different parts of this plant has never been reported for cancer treatment, especially compared to the anticancer effect of each part of *P. macrocarpa*. The current study also suggested that inducing apoptotic cells by different parts of *P. macrocarpa* could be more significant in determining which more potent extract to inhibit the growth of human breast cancer cells. The study revealed that ethanol extract of *P. macrocarpa* leaves triggers more apoptotic cell death than another plant part of *P. macrocarpa* in T47D cells.

In this study, the antiproliferative effect of various parts of *P. macrocarpa* leaves was indicated by a high increase of sub-G1 and decrease of S phase cells, which were mostly observed at IC_50_ concentration than high concentration. The percentage of cell division was also significantly reduced after treatment with *P. macrocarpa* leaves at dose-dependent manner for 48 h. These effects may be caused by several active compounds in the leaves of *P. macrocarpa*. Christina et al. [15] identified several bioactive compounds contained in *P. macrocarpa* leaf extract that contributed to the cancer regulation, including glycitein, corymboside, apigenin, sakuranetin, and sterubin (flavonoid group); betulin, cafestol, ageratriol, and carvone (terpenoid); sesamin and matairesinol (lignan); and fraxetin (coumarin). Based on the PASS server screening, among these compounds, corymboside has the highest activity in enhancing TP53 expression with Pa = 0.941. Betulin and apigenin also have high activity in triggering the activity of caspase-3 and apoptosis agonists, respectively (Pa = 0.974 and Pa = 0.847), compared to other obtained compounds [15]. For corymboside, there are no in vitro and in vivo studies regarding its efficacy on cancer cells. According to Anaya-Eugenio et al. [16], betulin is a pentacyclic triterpenoid compound that inhibits the growth of MDA-MB-231 cells downregulating NF-*κ*B p65 expression in silico and in vitro. In vitro experiment by Seo et al. [17] also confirmed that apigenin could decrease the colony formation of MCF-7 cells by downregulating the STAT3 signaling pathway.

Some studies also mention that this plant has a strong antioxidant effect on leaf and fruit extracts because it contains a lot of flavonoids and phenolics [18]. Our previous study revealed that the crude extract of *P. macrocarpa* leaves contains a high phenolic and flavonoid content and antioxidant activity that may be contributed to its cytotoxicity effect on T47D cells [12]. The seed of this plant effectively induces a cytotoxicity effect on T47D cells, which is higher than mesocarp and pericarp extract [19]. *P. macrocarpa* leaves and fruit can be used for traditional treatments in some cancers such as breast cancer and brain tumors. The content of phalerin and gallic acid compounds is the compound that most acts as an anticancer in the plant crown God [20]. Semipolar methanol extract from *P. macrocarpa* has been shown to have anticancer effects on breast cancer cells in 3 ways, including anti-proliferation, anti-angiogenic, and apoptotic induction [10].

Apoptosis plays a crucial role in killing cancer cells which are maintained by several gene-regulated cell deaths [21]. The apoptotic cells are characterized by several morphological changes such as chromosome condensation, DNA fragmentation, cell shrinkage, and induction of the apoptotic body [22]. The present study revealed that *P. macrocarpa* leaf extract induces apoptosis of T47D cells, indicated by the high level of cells undergoing late and early apoptosis, DNA fragmentation induction, and apoptotic features. Apoptosis induced by extrinsic and intrinsic pathways is regulated by caspase cascade and mitochondrial-dependent, respectively. Then, both pathways will cause the activation of caspase-3 and then changes the morphology of the cell to the apoptosis feature [23].

Bcl-2 is an anti-apoptotic protein encoded by the Bcl-2 gene that acts as a checkpoint in the regulation of apoptosis, while Bax is a pro-apoptotic protein from the Bcl-2 gene family [22]. Caspase-3 is also important for triggering apoptosis [24]. Compared to the untreated control group, the relative number of caspase-3 and Bax/Bcl-2 ratios raised significantly in *P. macrocarpa* leaf treatment group in a dose-dependent manner. Flow cytometry analysis also showed a decrease in all dose treatments of *P. macrocarpa* leaves. Furthermore, the decreased expression of caspase-3 in the *P. macrocarpa* leaf extract was superior to that in the cisplatin group. This finding is supported by our previous in silico study, which investigated the molecular mechanism of *P. macrocarpa* leaf extracts in killing breast cancer. The in silico study predicted that the sesamin compound of *P. macrocarpa* leaves effectively stimulated caspase-3 and Bax protein, while corymboside strongly inhibited Bcl-2 protein [15]. These results demonstrated that *P. macrocarpa* leaf extract induces apoptosis through mitochondrial disruption by changing the expression level of Bcl-2 and Bax and triggering the activation of caspase-3 in vitro.

## 5. Conclusion

Among three plant parts of *P. macrocarpa,* the leaf extract exhibited the most potent anticancer activity with selectivity only to T47D cells. *P. macrocarpa* leaves effectively induced apoptosis, inhibited proliferation, and arrested the cell cycle of T47D cells. The mechanism of anticancer activity might be influenced by the downregulation of Bcl-2 and upregulation of caspase-3 and Bax/Bcl-2 ratio. These findings suggested that *P. macrocarpa* leaf extract might become a highly promising agent for breast cancer treatment. Additional research is required to confirm the anticancer effect of *P. macrocarpa* leaf extracts in vivo.

## Figures and Tables

**Table 1:** IC_50_ of various parts of *P. macrocarpa*.

Sample	IC_50_ value (*μ*g/mL ± SD)		Selectivity index (SI)	SI activity
	T47D	TIG		
Pericarp	125 ± 2.33	594 ± 2.77	4.7	High selectivity
Mesocarp	144 ± 1.18	472 ± 2.67	3.3	High selectivity
Seed	139 ± 1.14	538 ± 2.73	3.9	High selectivity
Leaves	97 ± 1.82	414 ± 2.62	4.3	High selectivity

Data are expressed as mean ± SD. SI (selectivity index) = IC50 on normal cell line/IC50 on cancer cell line. SI value >3 indicated a high selective extract. Data are presented as mean ± SD of three independent experiments.

## Data Availability

The data used to support the findings of this study are included within the article.
